# 3-(2-Bromo­phen­yl)-*N*-phenyl­oxirane-2-carboxamide

**DOI:** 10.1107/S1600536809045929

**Published:** 2009-11-04

**Authors:** Long He, Hong-Mei Qin, Lian-Mei Chen

**Affiliations:** a College of Chemistry and Chemical Engineering, China West Normal University, Nanchong 637002, People’s Republic of China; b College of Life Science, China West Normal University, Nanchong 637002, People’s Republic of China

## Abstract

In the mol­ecule of the title compound, C_15_H_12_BrNO_2_, the two benzene rings adopt a *syn* configuration with respect to the ep­oxy ring; the dihedral angles between the ep­oxy ring and the two benzene rings are 59.90 (13) and 68.01 (12)°. Inter­molecular N—H⋯O and C—H⋯O hydrogen bonding is present in the crystal structure.

## Related literature

For epoxide-containing compounds used as building blocks in synthesis, see: Flisak *et al.* (1993 ▶); Watanabe *et al.* (1998 ▶); Zhu & Espenson (1995 ▶). For related structures, see: He (2009 ▶); He & Chen (2009 ▶).
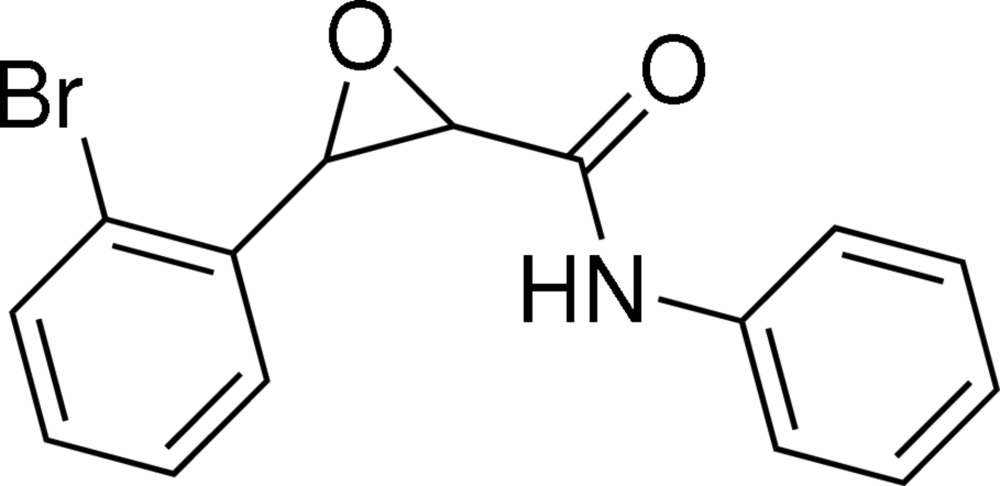


## Experimental

### 

#### Crystal data

C_15_H_12_BrNO_2_
*M* *_r_* = 318.17
Orthorhombic, 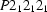

*a* = 6.71700 (10) Å
*b* = 10.0370 (2) Å
*c* = 20.4287 (3) Å
*V* = 1377.27 (4) Å^3^
*Z* = 4
Cu *K*α radiation
μ = 4.05 mm^−1^
*T* = 295 K
0.40 × 0.40 × 0.36 mm

#### Data collection

Oxford Diffraction Gemini S Ultra diffractometer
Absorption correction: multi-scan (*CrysAlis Pro*; Oxford Diffraction, 2009 ▶) *T*_min_ = 0.294, *T*_max_ = 0.324
17721 measured reflections
2701 independent reflections
2675 reflections with *I* > 2σ(*I*)
*R*_int_ = 0.028

#### Refinement

*R*[*F* ^2^ > 2σ(*F* ^2^)] = 0.028
*wR*(*F* ^2^) = 0.069
*S* = 1.01
2701 reflections
177 parameters
H atoms treated by a mixture of independent and constrained refinement
Δρ_max_ = 0.33 e Å^−3^
Δρ_min_ = −0.42 e Å^−3^
Absolute structure: Flack (1983 ▶), 1104 Friedel pairs
Flack parameter: −0.008 (18)

### 

Data collection: *CrysAlis Pro* (Oxford Diffraction, 2009 ▶); cell refinement: *CrysAlis Pro*; data reduction: *CrysAlis Pro*; program(s) used to solve structure: *SHELXS97* (Sheldrick, 2008 ▶); program(s) used to refine structure: *SHELXL97* (Sheldrick, 2008 ▶); molecular graphics: *ORTEP-3* (Farrugia, 1997 ▶); software used to prepare material for publication: *SHELXL97*.

## Supplementary Material

Crystal structure: contains datablocks global, I. DOI: 10.1107/S1600536809045929/xu2664sup1.cif

Structure factors: contains datablocks I. DOI: 10.1107/S1600536809045929/xu2664Isup2.hkl

Additional supplementary materials:  crystallographic information; 3D view; checkCIF report

## Figures and Tables

**Table 1:** Hydrogen-bond geometry (Å, °)

*D*—H⋯*A*	*D*—H	H⋯*A*	*D*⋯*A*	*D*—H⋯*A*
N1—H1⋯O2^i^	0.79 (3)	2.22 (3)	2.971 (2)	161 (2)
C15—H15⋯O1^ii^	0.93	2.59	3.442 (3)	153

Symmetry codes: (i) 
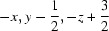
; (ii) 
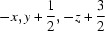
.

**Fig. 1.**
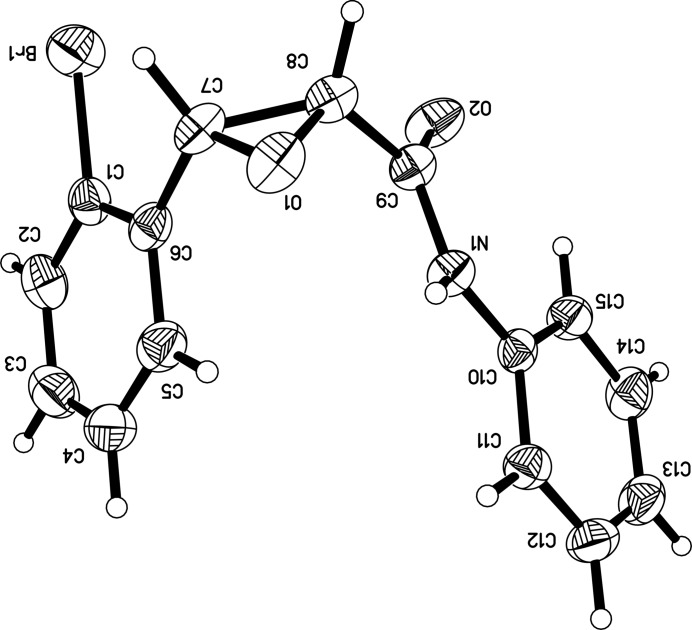
The molecular structure of (I) with 30% probability displacement ellipsoids (arbitrary spheres for H atoms).

## supplementary crystallographic information

### Comment

Epoxides are important intermediates in organic synthesis. Glycidic esters and amides are particularly useful as they can be further transformed to key intermediates of several pharmaceutical products (Flisak *et al.* 1993; Watanabe *et al.* 1998). The Darzens reaction, is one of the most powerful methodologies for the synthesis of α, β-epoxy carbonyl and related compounds (Zhu & Espenson, 1995). We report herein the crystal structure of the title compound.

The molecular structure of (I) is shown in Fig. 1. Bond lengths and angles in (I) are normal. In the molecule, the two phenyl ring adopts a *cis* configuration about the epoxides ring. The dihedral angle between the C1—C6 and C10—C15 is 77.05 (7)°, O1/C7/C8 epoxide ring makes dihedral angles of 59.90 (13)° and 68.01 (12)° with C6 and C15 phenyl ring, respectively, which is similar to that found in a related structure (He & Chen, 2009). The crystal packing is stabilized by N—H···0 and C—H···0 hydrogen bonding (Table 1).

### Experimental

2-Chloro-*N*-phenylacetamide (0.17 g, 1.0 mmol) and potassium hydroxide (0.112 g, 2.0 mmol) were dissolved in acetonitrile (2 ml). To the solution was added 2-bromophenylaldehyde (0.15 g, 1.0 mmol) at 298 K, the solution was stirred for 60 min and removal of solvent under reduced pressure, the residue was purified through column chromatography. Single crystals suitable for X-ray diffraction were obtained by slow evaporation of an ethyl acetate solution at room temperature for 1 d.

### Refinement

Imine H atom was located in a difference Fourier map and refined isotropically. The carbon-bound hydrogen atoms were placed in calculated positions, with C—H = 0.93–0.98 Å, and refined using a riding model with *U*_iso_(H) =1.2*U*_eq_(C).

### Crystal data

**Table:**

C_15_H_12_BrNO_2_	*F*(000) = 640
*M**_r_* = 318.17	*D*_x_ = 1.534 Mg m^−^^3^
Orthorhombic, *P*2_1_2_1_2_1_	Cu *K*α radiation, λ = 1.54184 Å
Hall symbol: P 2ac 2ab	Cell parameters from 15647 reflections
*a* = 6.7170 (1) Å	θ = 2.2–72.1°
*b* = 10.0370 (2) Å	µ = 4.05 mm^−^^1^
*c* = 20.4287 (3) Å	*T* = 295 K
*V* = 1377.27 (4) Å^3^	Block, colorless
*Z* = 4	0.40 × 0.40 × 0.36 mm

### Data collection

**Table:**

Oxford Diffraction Gemini S Ultra diffractometer	2701 independent reflections
Radiation source: Enhance Ultra (Cu) X-ray Source	2675 reflections with *I* > 2σ(*I*)
mirror	*R*_int_ = 0.028
Detector resolution: 15.9149 pixels mm^-1^	θ_max_ = 72.3°, θ_min_ = 4.3°
ω scans	*h* = −8→8
Absorption correction: multi-scan (*CrysAlis PRO*; Oxford Diffraction, 2009)	*k* = −11→12
*T*_min_ = 0.294, *T*_max_ = 0.324	*l* = −24→25
17721 measured reflections	

### Refinement

**Table:**

Refinement on *F*^2^	Hydrogen site location: inferred from neighbouring sites
Least-squares matrix: full	H atoms treated by a mixture of independent and constrained refinement
*R*[*F*^2^ > 2σ(*F*^2^)] = 0.028	*w* = 1/[σ^2^(*F*_o_^2^) + (0.035*P*)^2^ + 0.5235*P*] where *P* = (*F*_o_^2^ + 2*F*_c_^2^)/3
*wR*(*F*^2^) = 0.069	(Δ/σ)_max_ = 0.001
*S* = 1.00	Δρ_max_ = 0.33 e Å^−^^3^
2701 reflections	Δρ_min_ = −0.42 e Å^−^^3^
177 parameters	Extinction correction: *SHELXL97* (Sheldrick, 2008), Fc^*^=kFc[1+0.001xFc^2^λ^3^/sin(2θ)]^-1/4^
0 restraints	Extinction coefficient: 0.0074 (5)
Primary atom site location: structure-invariant direct methods	Absolute structure: Flack (1983), 1104 Friedel pairs
Secondary atom site location: difference Fourier map	Flack parameter: −0.008 (18)

### Special details

**Table:**

Geometry. All e.s.d.'s (except the e.s.d. in the dihedral angle between two l.s. planes) are estimated using the full covariance matrix. The cell e.s.d.'s are taken into account individually in the estimation of e.s.d.'s in distances, angles and torsion angles; correlations between e.s.d.'s in cell parameters are only used when they are defined by crystal symmetry. An approximate (isotropic) treatment of cell e.s.d.'s is used for estimating e.s.d.'s involving l.s. planes.
Refinement. Refinement of *F*^2^ against ALL reflections. The weighted *R*-factor *wR* and goodness of fit *S* are based on *F*^2^, conventional *R*-factors *R* are based on *F*, with *F* set to zero for negative *F*^2^. The threshold expression of *F*^2^ > σ(*F*^2^) is used only for calculating *R*-factors(gt) *etc*. and is not relevant to the choice of reflections for refinement. *R*-factors based on *F*^2^ are statistically about twice as large as those based on *F*, and *R*- factors based on ALL data will be even larger.

### Fractional atomic coordinates and isotropic or equivalent isotropic displacement parameters (Å^2^)

**Table:**

	*x*	*y*	*z*	*U*_iso_*/*U*_eq_	
Br1	−0.14003 (5)	0.63054 (3)	0.557209 (16)	0.07563 (15)	
O1	−0.1933 (3)	0.23595 (16)	0.66346 (9)	0.0567 (4)	
O2	−0.0059 (3)	0.50889 (16)	0.76057 (10)	0.0637 (5)	
N1	0.1077 (3)	0.29560 (18)	0.75313 (9)	0.0437 (4)	
C5	0.1656 (4)	0.2794 (3)	0.59008 (13)	0.0582 (6)	
H5	0.1480	0.2015	0.6140	0.070*	
C6	0.0186 (3)	0.3772 (2)	0.59138 (10)	0.0470 (5)	
C15	0.3410 (4)	0.4080 (2)	0.82806 (11)	0.0520 (5)	
H15	0.2588	0.4823	0.8314	0.062*	
C9	−0.0201 (3)	0.3947 (2)	0.74074 (11)	0.0440 (4)	
C8	−0.1951 (3)	0.3600 (2)	0.69813 (11)	0.0467 (5)	
H8	−0.3253	0.3893	0.7143	0.056*	
C10	0.2882 (3)	0.3016 (2)	0.78857 (10)	0.0407 (4)	
C7	−0.1724 (3)	0.3564 (2)	0.62606 (11)	0.0499 (5)	
H7	−0.2912	0.3828	0.6014	0.060*	
C11	0.4155 (3)	0.1931 (2)	0.78350 (11)	0.0494 (5)	
H11	0.3828	0.1227	0.7559	0.059*	
C1	0.0535 (4)	0.4931 (2)	0.55591 (11)	0.0502 (5)	
C12	0.5905 (4)	0.1889 (3)	0.81914 (14)	0.0607 (6)	
H12	0.6734	0.1149	0.8162	0.073*	
C2	0.2261 (5)	0.5119 (3)	0.52052 (13)	0.0654 (7)	
H2	0.2477	0.5908	0.4977	0.078*	
C3	0.3657 (5)	0.4116 (3)	0.51958 (14)	0.0723 (7)	
H3	0.4815	0.4227	0.4952	0.087*	
C13	0.6420 (4)	0.2934 (3)	0.85879 (13)	0.0655 (7)	
H13	0.7594	0.2905	0.8829	0.079*	
C14	0.5199 (4)	0.4020 (3)	0.86281 (13)	0.0641 (7)	
H14	0.5567	0.4734	0.8892	0.077*	
C4	0.3375 (4)	0.2965 (3)	0.55369 (14)	0.0680 (7)	
H4	0.4334	0.2297	0.5525	0.082*	
H1	0.074 (3)	0.225 (3)	0.7406 (12)	0.037 (6)*	

### Atomic displacement parameters (Å^2^)

**Table:**

	*U*^11^	*U*^22^	*U*^33^	*U*^12^	*U*^13^	*U*^23^
Br1	0.0896 (2)	0.05464 (17)	0.0827 (2)	0.01486 (15)	0.01432 (17)	0.01807 (14)
O1	0.0611 (10)	0.0400 (8)	0.0689 (10)	−0.0133 (7)	−0.0136 (8)	0.0087 (7)
O2	0.0677 (10)	0.0342 (8)	0.0891 (12)	0.0036 (8)	−0.0200 (10)	−0.0011 (8)
N1	0.0487 (10)	0.0311 (8)	0.0515 (9)	−0.0032 (8)	−0.0056 (8)	−0.0002 (7)
C5	0.0618 (15)	0.0547 (13)	0.0581 (13)	0.0042 (12)	−0.0126 (12)	−0.0037 (11)
C6	0.0500 (11)	0.0458 (11)	0.0453 (10)	−0.0031 (11)	−0.0122 (8)	−0.0024 (9)
C15	0.0584 (13)	0.0455 (11)	0.0520 (11)	0.0046 (10)	−0.0047 (11)	−0.0052 (9)
C9	0.0473 (10)	0.0332 (10)	0.0515 (11)	−0.0039 (8)	−0.0011 (9)	0.0071 (8)
C8	0.0418 (10)	0.0375 (10)	0.0609 (12)	−0.0063 (9)	−0.0036 (8)	0.0089 (10)
C10	0.0442 (10)	0.0386 (10)	0.0392 (9)	−0.0030 (8)	0.0020 (8)	0.0079 (8)
C7	0.0459 (11)	0.0448 (11)	0.0589 (12)	−0.0055 (10)	−0.0150 (9)	0.0106 (10)
C11	0.0517 (12)	0.0425 (11)	0.0541 (12)	0.0011 (9)	0.0042 (9)	0.0029 (9)
C1	0.0604 (12)	0.0462 (11)	0.0441 (10)	−0.0012 (9)	−0.0009 (10)	−0.0020 (10)
C12	0.0477 (12)	0.0615 (14)	0.0730 (16)	0.0100 (11)	0.0029 (11)	0.0138 (12)
C2	0.0782 (17)	0.0652 (16)	0.0528 (13)	−0.0086 (14)	0.0095 (12)	−0.0001 (12)
C3	0.0631 (16)	0.091 (2)	0.0629 (15)	−0.0026 (16)	0.0099 (14)	−0.0143 (14)
C13	0.0525 (13)	0.0838 (19)	0.0602 (13)	0.0019 (15)	−0.0100 (12)	0.0111 (13)
C14	0.0696 (16)	0.0679 (16)	0.0548 (13)	−0.0056 (14)	−0.0149 (12)	−0.0083 (12)
C4	0.0624 (15)	0.0748 (17)	0.0669 (15)	0.0141 (13)	−0.0095 (15)	−0.0156 (14)

### Geometric parameters (Å, °)

**Table:**

Br1—C1	1.896 (2)	C8—H8	0.9800
O1—C8	1.433 (3)	C10—C11	1.388 (3)
O1—C7	1.437 (3)	C7—H7	0.9800
O2—C9	1.219 (3)	C11—C12	1.384 (4)
N1—C9	1.338 (3)	C11—H11	0.9300
N1—C10	1.414 (3)	C1—C2	1.380 (4)
N1—H1	0.78 (3)	C12—C13	1.370 (4)
C5—C4	1.384 (4)	C12—H12	0.9300
C5—C6	1.392 (3)	C2—C3	1.375 (5)
C5—H5	0.9300	C2—H2	0.9300
C6—C1	1.390 (3)	C3—C4	1.363 (5)
C6—C7	1.481 (3)	C3—H3	0.9300
C15—C10	1.385 (3)	C13—C14	1.366 (4)
C15—C14	1.397 (4)	C13—H13	0.9300
C15—H15	0.9300	C14—H14	0.9300
C9—C8	1.503 (3)	C4—H4	0.9300
C8—C7	1.481 (3)		
			
C8—O1—C7	62.11 (14)	O1—C7—H7	114.9
C9—N1—C10	127.97 (19)	C6—C7—H7	114.9
C9—N1—H1	115.1 (18)	C8—C7—H7	114.9
C10—N1—H1	116.8 (18)	C12—C11—C10	120.6 (2)
C4—C5—C6	121.0 (3)	C12—C11—H11	119.7
C4—C5—H5	119.5	C10—C11—H11	119.7
C6—C5—H5	119.5	C2—C1—C6	121.9 (2)
C1—C6—C5	117.4 (2)	C2—C1—Br1	118.96 (19)
C1—C6—C7	120.9 (2)	C6—C1—Br1	119.09 (17)
C5—C6—C7	121.7 (2)	C13—C12—C11	120.1 (2)
C10—C15—C14	118.9 (2)	C13—C12—H12	119.9
C10—C15—H15	120.6	C11—C12—H12	119.9
C14—C15—H15	120.6	C3—C2—C1	118.7 (3)
O2—C9—N1	125.9 (2)	C3—C2—H2	120.6
O2—C9—C8	118.1 (2)	C1—C2—H2	120.6
N1—C9—C8	116.05 (19)	C4—C3—C2	121.2 (3)
O1—C8—C7	59.09 (14)	C4—C3—H3	119.4
O1—C8—C9	118.74 (18)	C2—C3—H3	119.4
C7—C8—C9	120.07 (19)	C14—C13—C12	119.7 (2)
O1—C8—H8	115.7	C14—C13—H13	120.2
C7—C8—H8	115.7	C12—C13—H13	120.2
C9—C8—H8	115.7	C13—C14—C15	121.3 (2)
C15—C10—C11	119.4 (2)	C13—C14—H14	119.3
C15—C10—N1	123.5 (2)	C15—C14—H14	119.3
C11—C10—N1	117.1 (2)	C3—C4—C5	119.7 (3)
O1—C7—C6	117.2 (2)	C3—C4—H4	120.1
O1—C7—C8	58.80 (14)	C5—C4—H4	120.1
C6—C7—C8	124.15 (18)		
			
C4—C5—C6—C1	−1.3 (3)	C9—C8—C7—O1	107.5 (2)
C4—C5—C6—C7	175.4 (2)	O1—C8—C7—C6	−103.6 (2)
C10—N1—C9—O2	−3.1 (4)	C9—C8—C7—C6	3.9 (4)
C10—N1—C9—C8	176.5 (2)	C15—C10—C11—C12	2.1 (3)
C7—O1—C8—C9	−109.7 (2)	N1—C10—C11—C12	−176.9 (2)
O2—C9—C8—O1	167.2 (2)	C5—C6—C1—C2	0.1 (3)
N1—C9—C8—O1	−12.5 (3)	C7—C6—C1—C2	−176.7 (2)
O2—C9—C8—C7	98.3 (3)	C5—C6—C1—Br1	−178.98 (16)
N1—C9—C8—C7	−81.4 (3)	C7—C6—C1—Br1	4.2 (3)
C14—C15—C10—C11	−1.2 (3)	C10—C11—C12—C13	−1.4 (4)
C14—C15—C10—N1	177.8 (2)	C6—C1—C2—C3	1.1 (4)
C9—N1—C10—C15	14.1 (3)	Br1—C1—C2—C3	−179.8 (2)
C9—N1—C10—C11	−166.9 (2)	C1—C2—C3—C4	−1.2 (4)
C8—O1—C7—C6	115.3 (2)	C11—C12—C13—C14	−0.2 (4)
C1—C6—C7—O1	−177.83 (19)	C12—C13—C14—C15	1.1 (4)
C5—C6—C7—O1	5.5 (3)	C10—C15—C14—C13	−0.4 (4)
C1—C6—C7—C8	−108.6 (2)	C2—C3—C4—C5	0.0 (4)
C5—C6—C7—C8	74.7 (3)	C6—C5—C4—C3	1.3 (4)

### Hydrogen-bond geometry (Å, °)

**Table:**

*D*—H···*A*	*D*—H	H···*A*	*D*···*A*	*D*—H···*A*
N1—H1···O2^i^	0.79 (3)	2.22 (3)	2.971 (2)	161 (2)
C15—H15···O1^ii^	0.93	2.59	3.442 (3)	153

Symmetry codes: (i) −*x*, *y*−1/2, −*z*+3/2; (ii) −*x*, *y*+1/2, −*z*+3/2.
